# Repeated intravesical injections of platelet-rich plasma improve symptoms and alter urinary functional proteins in patients with refractory interstitial cystitis

**DOI:** 10.1038/s41598-020-72292-0

**Published:** 2020-09-16

**Authors:** Yuan-Hong Jiang, Yuh-Chen Kuo, Jia-Fong Jhang, Cheng-Ling Lee, Yung-Hsiang Hsu, Han-Chen Ho, Hann-Chorng Kuo

**Affiliations:** 1 Department of Urology, Hualien Tzu Chi Hospital, Buddhist Tzu Chi Medical Foundation, and Tzu Chi University, Hualien, Taiwan; 2 Department of Urology, Yangming Branch of Taipei City Hospital, Taipei, Taiwan; 3 Department of Pathology, Hualien Tzu Chi Hospital, Buddhist Tzu Chi Medical Foundation, and Tzu Chi University, Hualien, Taiwan; 4 Department of Anatomy, Tzu Chi University, Hualien, Taiwan

**Keywords:** Neurophysiology, Urogenital diseases

## Abstract

Repeated intravesical injections of autologous platelet-rich plasma (PRP) have been shown to improve symptoms in patients with interstitial cystitis/bladder pain syndrome (IC/BPS); however, there is a paucity of objective evidence of the effectiveness of this therapy. In this study, we investigated the changes in urinary markers after PRP treatment. Forty patients with IC/BPS who were refractory to conventional therapy received four injections of PRP at monthly intervals; 10 mL PRP solution with 2.5 times the peripheral blood platelet concentration was used. Urine levels of thirteen functional proteins, growth factors, and cytokines were assessed at baseline and at the 4th PRP injection. The clinical parameters included visual analog scale (VAS) pain score, daily urinary frequency, nocturia episodes, functional bladder capacity, and global response assessment (GRA). The GRA and symptom score significantly decreased post-treatment. In patients with GRA ≥ 2, the success rates at 1 month and at 3 months after the 4th PRP injection were 70.6% and 76.7%, respectively. The VAS pain score, frequency, and nocturia showed a significant decrease (all *p* < 0.05). Urinary levels of nerve growth factor, matrix metalloproteinase-13, and vascular endothelial growth factor significantly decreased post-treatment (*p* = 0.043, *p* = 0.02, and *p* = 0.000, respectively); platelet-derived growth factor-AB showed a significant increase (*p* = 0.004) at the 4th PRP treatment compared with baseline. In this study, repeated intravesical PRP injections provided significant symptom improvement in IC/BPS patients with concomitant changes in the related biomarker levels.

Trial registration: ClinicalTrial.gov: NCT03104361; IRB: TCGH 105-48-A.

## Introduction

Interstitial cystitis/bladder pain syndrome (IC/BPS) is a chronic bladder inflammatory disease characterized by frequency and bladder pain. According to previous studies, the pathophysiology of IC/BPS includes urothelial abnormalities, afferent sensory nerve activation, and mast cell over-activation in the bladder^1,2^. The available evidence suggests that IC/BPS is caused by chronic unresolved bladder inflammation and subsequent development of urothelial dysfunction and interstitial neurogenic inflammation^3^. Resolution of bladder inflammation is essential to achieve successful outcomes of treatment targeting the urothelial barrier dysfunction or pain.

The treatment modalities for IC/BPS are not well-standardized. The conventional therapies include cystoscopic hydrodistention, oral pentyosan polysulphate, chondroitin sulphate, intravesical instillation of hyaluronic acid, non-steroid anti-inflammatory drugs, intravesical injection of botulinum toxin A, sacral neuromodulation, and psychotherapy. Most treatment modalities are not backed by scientific evidence from clinical trials; in addition, patients are usually treated empirically depending on the severity of symptoms. Currently, no definitive therapy has been shown to provide successful long-term outcomes^2^.

The most common clinical presentation of IC/BPS is bladder and pelvic pain, glomerulations under cystoscopic hydrodistention, and denudation or thinning of the bladder epithelium^1,2^. Previous studies have documented increased urinary and tissue expressions of neurotrophins such as nerve growth factor (NGF) and brain-derived neurotrophic factor (BDNF); this indicates that sensory nerve activation and neural plasticity may account for the increased urinary frequency and bladder pain in these patients^4,5^. Elevated urinary levels of NGF and BDNF are associated with sensory activation and increased pain perception in IC/BPS^6^. In addition, studies have documented reduction in urinary NGF level after effective treatment of IC/BPS^7^.

Our recent study suggested that increased apoptosis in bladder urothelium of patients with IC/PBS may be attributable to the upregulation of inflammatory signals^8^. Chronic inflammatory diseases are typically associated with increased levels of inflammatory cytokines, such as tumor necrosis factor (TNF)-α, interleukin (IL)-6, and IL-8^9^. Binding of TNF-α to the TNF-receptor 1 has been shown to induce inflammation and upregulation of apoptosis^10^. Chronic inflammation may mediate apoptosis and alter the normal urothelial cell homeostasis upon bladder insult; this impairs the integrity of the urothelial umbrella cells and deranges the barrier function^11^.

Glomerulations during hydrodistention exhibit a strong association with the overexpression of angiogenic growth factors, such as platelet-derived endothelial cell growth factor (PDGF), in the bladder. Neovascularization promoted by angiogenic growth factors likely plays an important role in the pathogenesis of IC/PBS, inducing glomerulations during hydrodistension^12^. Patients with IC/PBS exhibit increased expression of vascular endothelial growth factor (VEGF) and immature vascularization; in addition, VEGF overexpression is associated with the degree of urinary frequency and bladder pain in patients with IC/BPS^13^. Collectively, these findings indicate that VEGF may contribute to pain and promote the formation of immature vessels in IC/PBS; moreover, enhanced immature vascularization may play a role in inducing glomerulations in these patients^14^.

In patients with chronic IC/BPS patients, bladder fibrosis may also contribute to reduced bladder capacity during hydrodistention. Moreover, bladder fibrosis is usually associated with reduced functional bladder capacity (FBC) and increased bladder pain and IC symptoms. Transforming growth factor beta 1 (TGF-β1) has been implicated in the causation of bladder fibrosis after organ or tissue injury^15^. Upregulation of TGF-β has been shown to induce nerve hyperplasia and fibrosis in ketamine-associated cystitis^16^. Matrix metalloproteinases (MMPs) are involved in physiological tissue remodeling. In addition, high expression levels of MMP have been documented in pathological conditions such as human carcinoma and inflammatory diseases. MMPs can degrade certain components of the extracellular matrix and may be associated with inflammation; moreover, MMPs also play a role in tissue fibrosis^17^. Thus, MMPs may also be involved in reducing bladder capacity in IC/BPS through inflammatory pathways.

Recently, autologous platelet-rich plasma (PRP) has been widely applied as a regenerative therapy to facilitate wound healing and to hasten recovery from injuries^18^. Platelets can release several growth factors, cytokines, and modulators of the extracellular matrix; in addition, these modulate the wound healing process including inflammation and tissue regeneration^19^. PRP has been widely used in the treatment of orthopedic and soft-tissue inflammation; however, their role in the treatment of bladder disorders is not well characterized. In our previous pilot study, repeated intravesical injections of PRP were found to alleviate the symptoms of IC^20,21^. However, clinical therapeutic efficacy of any novel treatment for IC/BPS is liable to be affected by subjective perception of improvement. Therefore, there is a need to obtain more robust evidence to prove its therapeutic efficacy. Since several pathological processes may be involved in the pathogenesis of IC/BPS, it will be rational to assess the changes in urinary functional proteins, growth factors, and cytokines related to chronic inflammation, fibrosis, angiogenesis, and tissue regeneration and growth after PRP injections. The results of this study may provide objective evidence of the therapeutic effects of PRP in patients with IC/BPS.

## Results

The mean age of 40 patients (37 females and 3 males) was 55.5 ± 11.1 years. All patients received four intravesical injections of PRP at monthly intervals. No patient with Hunner’s lesion was included in this study. The parameters at baseline, at the time of 4th PRP injection, and at 3 months after the 4th PRP injection are summarized in Table 1. Significant improvement in IC symptom index (ICSI), IC problem index (ICPI), O’Leary Sant symptom score (OSS), VAS, FBC, and GRA were noted at these time-points.

**Table 1:** Changes in the various parameters from baseline to the 4th PRP injection, and 3 months after the 4th PRP injection.

	(A) Baseline 1st PRP (n = 40)	(B) 4th-PRP (n = 40)	(C) 4th PRP-3M (n = 40)	*p* value
ICSI	9.04 ± 3.72	5.98 ± 3.72	5.03 ± 3.25	All *p* < 0.05
ICPI	9.80 ± 3.60	6.44 ± 4.07	4.23 ± 3.46	All *p* < 0.05
OSS	19.1 ± 6.95	12.4 ± 7.11	9.26 ± 6.33	All *p* < 0.05
VAS	3.38 ± 2.89	1.16 ± 1.64	1.10 ± 1.85	All *p* < 0.05
FBC (mL)	291 ± 134	318 ± 126	335 ± 98.3	A v C, *p* < 0.05
Frequency	14.6 ± 10.3	11.1 ± 5.31	9.97 ± 3.42	A v C, *p* < 0.05
Nocturia	2.60 ± 1.32	2.23 ± 1.33	1.97 ± 1.33	A v C, *p* < 0.05
Qmax (mL/s)	10.6 ± 7.84	11.2 ± 5.75	23.9 ± 12.4	A v C, *p* < 0.05
Volume (mL)	210 ± 122	229 ± 127	243 ± 105	All *p* > 0.05
PVR (mL)	66.4 ± 139	56.5 ± 103	15.4 ± 17.2	A v C, *p* < 0.05
CBC (mL)	274 ± 132	284 ± 123	259 ± 106	All *p* > 0.05
GRA	0	1.82 ± 0.98	1.94 ± 0.93	All *p* < 0.05

*ICSI* Interstitial Cystitis Symptom Index, *ICPI* Interstitial Cystitis Problem Index, *OSS* O”Leary-Sant symptom score, *VAS* visual analog score, *FBC* functional bladder capacity, *Qmax* maximum flow rate, *PVR* post-void residua, *CBC* cystometric bladder capacity, *GRA* Global response assessment, *PRP* platelet-rich plasma.

The GRA and symptom score significantly decreased after the 1st PRP injection and the therapeutic effect persisted up to 3 months after the 4th PRP injection. The success rate (defined as improvement in GRA by ≥ 2) was 45% at 1 month after the 1st PRP injection, 70% at the 4th PRP injection and at 1 month after the 4th PRP injection, and 67.5% at 3 months after the 4th PRP injection. There was significant decrease in OSS, ICSI, ICPI, and VAS pain score and significant improvement in GRA (*p* < 0.05 for all). After four PRP injections, there was a significant increase in the Qmax with no concomitant increase in the post-void residual (PVR) volume. The urodynamic parameters at baseline showed no significant difference from those at 3 months after the 4th PRP injection. However, 20/27 (74.1%) of patients with a successful result had a negative KCl test after PRP treatment, while 11/13 patients (84.6%) with a failed result continued to show positive KCl test after PRP treatment^21^.

At the 4th PRP injection time-point, urinary levels of NGF, MMP-13, and VEGF were significantly decreased (*p* = 0.043, *p* = 0.001, and *p* < 0.0001, respectively), and that of PDGF-AB were significantly increased (*p* = 0.004) compared with baseline (Table 2). On comparing the changes in these markers between patients with GRA ≥ 2 and GRA < 2, significant changes in NGF, PDGF, MMP-13, and VEGF were observed in patients with GRA ≥ 2, while significant changes in MMP-13, IL-6, and VEGF were observed in patients with GRA < 2 (Table 3). The urinary VEGF level was reduced in all patients after the 1st PRP injection and remained low at the 4th PRP time-point. None of the patients developed urinary tract infection or difficulty in micturition. No significant changes were observed with respect to other urinary proteins or cytokine levels after PRP injections. We also measured the urine markers in 10 women with stress urinary incontinence with no bladder symptoms, as a control group. Only urinary VEGF level was significantly higher in IC/BPS patients at baseline (17.7 ± 6.67 vs 9.45 ± 10.6, *p* = 0.004); however, it was significantly lower after PRP injection as compared to that in the controls (all *p* < 0.0001).

**Table 2:** Changes in urinary cytokines, functional proteins, and growth factors in IC/BPS patients treated with repeat platelet rich plasma injection.

	Baseline 1st PRP (n = 40)	1 M 2nd PRP (n = 40)	2 M 3rd PRP (n = 40)	3 M 4th PRP (n = 40)	*p* value BL v End point
NGF	0.29 ± 0.04	0.28 ± 0.12	0.30 ± 0.01	0.23 ± 0.16	0.043
BDNF	0.5 ± 0.24	–	–	0.5 ± 0.46	0.986
TGFβ1	12.4 ± 16.0	–	–	9.14 ± 9.28	0.315
PDGF-AA	36.1 ± 32.9	–	–	37.7 ± 28.4	0.758
PDGF-AB	2.78 ± 1.54	5.10 ± 7.37	5.10 ± 7.96	4.3 ± 2.91	0.004
MMP-13	27.3 ± 17.3	28.4 ± 6.56	21.6 ± 26.8	17.4 ± 3.98	0.001
MMP-1	22.3 ± 33.4	–	–	13.5 ± 17.7	0.111
IL-2	0.69 ± 0.25	0.69 ± 0.08	0.80 ± 0.31	0.66 ± 0.24	0.354
IL-6	2.67 ± 6.24	11.98 ± 61.5	1.38 ± 0.91	3.07 ± 6.22	0.295
IL-8	19.7 ± 33.5	44.1 ± 167	27.4 ± 75.2	27.0 ± 47.7	0.466
VEGF	17.7 ± 6.67	3.37 ± 4.54	3.51 ± 6.46	0.10 ± 0.26	0.000
IL-1β	0.9 ± 1.11	–	–	0.69 ± 0.21	0.285
TNFα	0.9 ± 0.86	0.9 ± 0.98	1.40 ± 2.63	1.4 ± 1.77	0.120

*PRP* platelet rich plasma, *NGF* nerve growth factor, *BDNF* brain-derived neurotrophic factor, *TGF-β1* transforming growth factor beta 1, *PDGF* platelet-derived growth factor, *MMP* matrix metalloproteinase, *IL* interleukin, *VEGF* vascular endothelial growth factor, *TNFα* tumor necrosis factor α.

**Table 3:** Changes in urinary cytokines, functional proteins, and growth factors in IC/BPS patients with successful and failed treatment with repeat platelet-rich plasma (PRP) injections.

	GRA ≥ 2 (n = 27)	GRA < 2 (n = 13)	*p* value	Δ*p* value
NGF_BL	0.29 ± 0.04	0.29 ± 0.04	0.911	0.428
NGF_Tx	0.21 ± 0.18	0.28 ± 0.12	0.174	
BDNF_BL	0.52 ± 0.28	0.45 ± 0.1	0.276	0.821
BDNF_Tx	0.48 ± 0.45	0.54 ± 0.5	0.656	
TGFB_BL	10.2 ± 12.2	18.0 ± 22.9	0.323	0.799
TGFB_Tx	7.67 ± 1.89	12.8 ± 17.2	0.370	
PDGF-AA_BL	31.2 ± 29.9	48.3 ± 38.2	0.148	0.832
PDGF-AA_Tx	32.1 ± 25.1	51.8 ± 32.4	0.062	
PDGF-AB_BL	2.44 ± 0.9	3.61 ± 2.39	0.150	0.534
PDGF-AB_Tx	4.15 ± 3.12	4.64 ± 2.44	0.661	
MMP-13_BL	28.5 ± 20.2	24.0 ± 4.65	0.426	0.251
MMP-13_Tx	16.4 ± 3.83	19.5 ± 3.68	0.040	
MMP-1_BL	25.3 ± 39.1	14.4 ± 5.86	0.399	0.530
MMP-1_Tx	14.3 ± 20.5	11.0 ± 6.29	0.625	
IL-2_BL	0.74 ± 0.28	0.58 ± 0.10	0.012	0.620
IL-2_Tx	0.69 ± 0.26	0.58 ± 0.18	0.201	
IL-6_BL	3.43 ± 7.45	1.02 ± 0.69	0.231	0.842
IL-6_Tx	3.8 ± 7.42	1.49 ± 0.99	0.316	
IL-8_BL	23.7 ± 39.1	10.8 ± 12.8	0.216	0.869
IL-8_Tx	32.3 ± 56.6	15.2 ± 10.8	0.334	
VEGF_BL	18.5 ± 7.15	15.7 ± 5.27	0.170	0.157
VEGF_Tx	0.1 ± 0.31	0.09 ± 0.13	0.904	
IL-1β_BL	0.95 ± 1.21	0.77 ± 0.85	0.655	0.833
IL-1β_Tx	0.72 ± 0.24	0.63 ± 0.06	0.101	
TNFα_BL	0.97 ± 0.97	0.74 ± 0.05	0.543	0.658
TNFα_Tx	1.57 ± 2.08	1.01 ± 0.24	0.415	

*GRA* global response assessment, *BL* baseline, *Tx* post-PRP treatment, *PRP* platelet rich plasma, *NGF* n*erve growth factor*, *BDNF* brain-derived neurotrophic factor, *TGF-β1* transforming growth factor beta 1, *PDGF* platelet-derived growth factor, *MMP* matrix metalloproteinase, *IL* interleukin, *VEGF* vascular endothelial growth factor, *TNFα* tumor necrosis factor α.

The changes in urinary marker levels and the concomitant improvement in the clinical symptoms of IC are shown in Table 4. The urinary NGF level decreased in patients with OSS < 10 after PRP treatment. The PDGF-AB level showed a significant increase with improvement in all clinical variables except in patients with FBC > 350 mL. The levels of MMP-13 and VEGF showed a significant decrease in association with improvement in all clinical variables. Interestingly, the urinary levels of VEGF and MMP-13 showed a significant decrease even in patients who failed to show improvement in clinical variables.

**Table 4:** Changes in urinary markers and their association with clinical improvement after PRP injections.

		N =	NGF	PDGF-AB	MMP-13	VEGF
GRA ≥ 2	BL	27	0.29 ± 0.04	2.44 ± 0.9	28.5 ± 20.2	18.5 ± 7.15
	Tx		0.21 ± 0.18*	4.15 ± 3.12*	16.4 ± 3.83*	0.06 ± 0.10*
GRA < 2	BL	13	0.29 ± 0.04	3.61 ± 2.39	24.0 ± 4.65	15.7 ± 5.27
	Tx		0.28 ± 0.12	4.64 ± 2.44	19.5 ± 3.68*	0.09 ± 0.13*
VAS ≤ 2	BL	32	0.28 ± 0.04	2.62 ± 1.17	25.7 ± 8.59	17.2 ± 6.22
	Tx		0.22 ± 0.16	3.91 ± 2.83*	17.3 ± 4.10*	0.09 ± 0.29*
VAS > 2	BL	18	0.30 ± 0.03	3.21 ± 2.44	27.4 ± 27.2	18.0 ± 7.43
	Tx		0.27 ± 0.11	5.38 ± 2.97	20.9 ± 35.3	0.09 ± 0.09*
Frequency ≤ 10/day	BL	22	0.30 ± 0.42	2.93 ± 1.25	26.3 ± 8.46	17.8 ± 6.40
	Tx		0.22 ± 0.18	4.52 ± 2.63*	18.1 ± 3.41*	0.15 ± 0.35*
Frequency > 10/day	BL	18	0.27 ± 0.03	2.65 ± 1.95	15.4 ± 26.7	17.9 ± 6.27
	Tx		0.25 ± 0.12	3.87 ± 3.50	20.9 ± 35.3	0.03 ± 0.04*
FBC ≥ 350 mL	BL	11	0.30 ± 0.02	2.78 ± 1.40	23.3 ± 7.65	17.9 ± 5.45
	Tx		0.22 ± 0.17	4.92 ± 3.20	17.9 ± 3.73*	0.21 ± 0.50*
FBC < 350 mL	BL	29	0.28 ± 0.04	2.83 ± 1.63	29.7 ± 19.9	17.8 ± 6.67
	Tx		0.24 ± 0.15	3.99 ± 2.95	17.7 ± 4.05*	0.06 ± 0.96*
OSS < 10	BL	19	0.30 ± 0.04	2.37 ± 0.81	24.9 ± 10.3	18.8 ± 6.16
	Tx		0.18 ± 0.18*	4.76 ± 3.75*	16.1 ± 4.01*	0.13 ± 0.37*
OSS > 10	BL	21	0.28 ± 0.04	3.03 ± 1.85	30.2 ± 20.9	16.1 ± 6.46
	Tx		0.28 ± 0.11	3.87 ± 2.09*	18.3 ± 3.76*	0.06 ± 0.10*

*Indicates significant difference between baseline (BL) and post-PRP treatment (Tx), *NGF* nerve growth factor, *PDGF* platelet-derived growth factor, *MMP* matrix metalloproteinase, *VEGF* vascular endothelial growth factor, *PRP* platelet rich plasma, *GRA* global response assessment, *VAS* visual analog symptom, *FBC* functional bladder capacity, *OSS* O’Leary Sant symptom score.

## Discussion

This study provided evidence of the changes in urinary protein expressions to support the therapeutic effects of PRP in the treatment of IC/BPS refractory to conventional therapies. The urine biomarkers such as NGF, PDGF-AB, MMP-13, and VEGF showed significant changes after three PRP injections. These findings indicate that PRP can alter the bladder environment and alleviate inflammation in the diseased bladders.

The clinical manifestations of IC reflect the inflammatory process and neural upregulation in the bladder wall^22^. The clinical presentation of IC/BPS is heterogeneous; patients may present with frequency, urgency, and/or bladder pain with or without somatic symptoms^23^. In our previous proteomic study, we investigated the markers of inflammation, angiogenesis, and urothelial cell apoptosis in IC/BPS bladder and urine. The findings revealed the involvement of several signal transduction pathways in the pathophysiology of IC/BPS^24^. The elevated protein expressions in IC/BPS bladders showed a decrease after intravesical injection of botulinum toxin A, including VEGF, platelet factor 4, IL-1β, IL-8, CXCL16, and TIMP-4; these findings reflect the changes in bladder condition after effective treatment^24^. However, in this study, none of the functional proteins or cytokine levels (BDNF, TGFβ1, IL-2, I-6, IL-8, IL-1β, TNFα) showed a decrease after PRP treatment, with the exception of urinary NGF. This indicates that the chronic inflammation may not have been adequately resolved by PRP treatment. More injections or a higher concentration of PRP may be necessary to achieve adequate anti-inflammatory effect.

The mechanism of the therapeutic effect of intravesical PRP injection in patients with IC/BPS has not been clearly elucidated. In previous studies, PRP was shown to improve urinary frequency, urgency, and bladder pain^20,21^. Interestingly, the positive potassium test at baseline urodynamic study turned negative in 20 out of 27 patients with a successful result; however, 11 out of 13 patients with a failure result remained positive^21^. The potassium test assesses the increased urothelial permeability which is highly prevalent in patients with IC/BPS, bacterial cystitis, and irradiation cystitis^25^. The effect of PRP on urothelial barrier deficit indicated its beneficial effect in alleviating bladder inflammation and urothelial dysfunction.

VEGF is a major regulator of physiological or pathological angiogenesis^26^. Patients with IC/BPS were shown to exhibit significantly higher expressions of VEGF in the bladder urothelium; in addition, the expression level was associated with the grade of bladder pain^14^. In a previous study, injection of botulinum toxin A was shown to reduce the expression of VEGF in the bladder tissue of patients with IC/BPS; this was associated with a concomitant decrease in inflammatory marker levels in bladder^27^. Anti-VEGF neutralizing antibodies were shown to reduce pelvic/bladder pain in the cyclophosphmide cystitis model of bladder pain^28^. In the present study, post-treatment reduced expressions of VEGF were observed both in patients with GRA ≥ 2 and those with GRA < 2; this suggests that PRP has a therapeutic inhibitory effect on aberrant vascular angiogenesis of IC/BPS which is associated with a decrease in urothelial permeability.

PDGF is one of the several growth factors that regulate cell growth and division. PDGF is a potent mitogen that stimulates differentiation of mesenchymal cells into fibroblasts, smooth muscle cells, and glioblasts; PDGF is essential for wound healing and growth of blood vessels^29^. Sacral neuromodulation in patients with IC/BPS was shown to decrease the urinary PDGF level^30^. After PRP injections into the suburothelium, there was a significant increase in the urinary level of PDGF. In this study, we found significantly increased PDGF-AB level in patients with greater VAS pain improvement, less frequency, reduced IC symptoms, and those with GRA ≥ 2; these findings suggest that PRP injection may induce repair of the defective urothelim by enhancing the growth of fibroblasts, smooth muscle cells, and glial cells.

No previous studies have documented the role of MMP-13 in IC/BPS. MMPs are highly overexpressed in pathological conditions such as human carcinoma or inflammatory diseases. Imbalance of MMPs may play a role in tissue fibrosis^17^. Therefore, MMPs may affect the bladder capacity in IC/BPS by enhancing the inflammatory pathway. In this study, PRP injections reduced the overexpression of MMP-13 in patients with both GRA ≥ 2 and GRA < 2. In addition, the MMP-13 expression was significantly lower in patients with decreased urinary frequency and GRA ≥ 2, which suggests an association between reduction in MMP-13 expression and alleviation of inflammation after repeated PRP injections.

The changes in urinary NGF, VEGF, MMP-13, and PDGF-AB levels after intravesical PRP injections provide objective evidence of the role of PRP in alleviating inflammation, reducing abnormal angiogenesis, and improving the regeneration of defective urothelium in IC/BPS bladder. The condition of the bladder tends to vary in patients with IC/BPS; therefore, patients may exhibit variable response to treatment with respect to inflammation, angiogenesis, and tissue regeneration. Urinary biomarker levels may not entirely reflect the true inflammatory condition in the bladder; however, the significant changes in these urinary biomarkers after PRP treatment are, at least in part, associated with decreased bladder pain, decreased frequency, decreased clinical indices, and increased GRA. In addition, the high rate of negative KCl test after PRP treatment indicates that PRP injection may provide an opportunity for restoration of normal homeostasis in the urothelium of patients with IC/BPS. Although further studies are required to determine the optimal duration of PRP therapy, this treatment provides a safe therapeutic alternative for IC/BPS refractory to conventional therapies.

Since this is a pilot study, several critical points need to be addressed. In this study, the mean concentration of PRP was only 2.5 times the peripheral platelet count. Several studies have shown that a PRP concentration of 5 to 7.5 times shows the best therapeutic efficacy^31^. Previous studies of PRP in the context of osteoarthritis, fasciitis, and chronic ulcer entailed PRP injection directly into the wound with good therapeutic effect. However, the IC/BPS bladder is affected by inflammation throughout the bladder wall; therefore, local injection of PRP may not be effective in all patients. Secondly, the plasma added to the PRP may have contained anti-platelet factors that inhibit the activation of platelets. In an animal study, addition of normal saline to the PRP showed a significant therapeutic effect on angiogenesis in the infected wound^32^. Furthermore, IC/BPS patients tend to have unrealistic expectations from any new therapeutic modality; however, good treatment outcomes were observed in this study. Nonetheless, several aspects of PRP therapy and its modalities need to be standardized. These include the evaluation of potential placebo effect and determination of the optimal concentration of PRP, the injection sites, and the added solution. Therefore, more work is required before widespread clinical application of this regenerative treatment for IC/BPS.

In conclusion, this study demonstrated the safety and efficacy of intravesical injections of autologous PRP in alleviating IC symptoms. The significant decrease in the urinary NGF, MMP-13, and VEGF levels and the increase in PDGF-AB level after repeated PRP injections in patients with GRA ≥ 2 provide evidence of the therapeutic efficacy in patients with IC/BPS. The decrease in certain inflammation-related proteins after PRP injections suggest that the symptomatic improvement was likely attributable to the anti-inflammatory effect of PRP injections.

## Materials and methods

The clinical data were harvested from our recent clinical trial in which 40 patients with IC/BPS received repeated PRP injections at 1-month intervals^21^. All patients had been previously treated with at least three conventional therapeutic modalities including instillation of hyaluronic acid and botulinum toxin A injection; however, the symptoms of IC had flared up or failed to resolve. The minimum time elapsed since the most recent botulinum toxin A injection was one year before this treatment.

All patients were thoroughly investigated previously by cystoscopic hydrodistention, uroflowmetry, urodynamic study, 3-day voiding diary, visual analog pain scale (VAS) score, and OSS questionnaire to confirm the presence of IC and exclude patients with Hunner’s lesion, detrusor overactivity, detrusor underactivity with large PVR volume, or bladder outlet obstruction. Four treatments were administered to each patient and the therapeutic outcome was evaluated by the changes in OSS, including ICSI and ICPI. This study was approved by the Ethics Committee of the Buddhist Tzu Chi General Hospital*.* Each patient was informed about the study rationale and procedures, and written informed consent was obtained prior to treatment. All methods were performed in accordance with the relevant guidelines and regulations.

The detailed procedure for the preparation of PRP is described elsewhere^21^. In the morning of PRP injection, 50 mL of whole blood was withdrawn by a nurse and PRP was prepared in the central laboratory of the hospital. A licensed medical technologist centrifuged the blood with a soft spin (190×*g*, 20 min, < 20 °C). The supernatant plasma which contained platelets was transferred into another sterile tube without disturbing the buffy coat (without anticoagulant). The platelet containing plasma was further centrifuged by a hard spin (2,000×*g*, 20 min, < 20 °C). The platelet pellets were formed at the bottom of the tube. The upper two thirds of plasma was platelet poor plasma (PPP). The PPP was removed and the platelet pellets were added to the plasma depending on the required PRP concentration. In this study, 10 mL sterile PRP was prepared by gently shaking the tube containing platelet pellets and 10 mL plasma. One mL of PRP was sent for bacterial culture and another 1 mL was used to determine the platelet count. The final concentration of PRP in this study was approximately 2.5 times the peripheral blood platelet count.

The injection was administered under intravenous general anesthesia. In each treatment session, the patients received 20 suburothelial injections of PRP solution to cover most areas of the bladder wall; 0.5 mL PRP was administered at each injection site. During the procedure, the bladder volume was maintained at 100 mL to facilitate needle injections. The injection was performed using a 23 gauge needle and rigid cystoscopic injection instrument (22 Fr, Richard Wolf, and Knittlingen, Germany). The needle was inserted about 1 mm into the suburothelium at the posterior and lateral walls. After the PRP injections, an indwelling 14 Fr urethral Foley catheter was retained overnight and patients were discharged on the next day. Oral antibiotics were prescribed for 3 days. All patients were followed up in the outpatient clinic every month and for 3 months after the 4th PRP treatment session^21^. The effect of PRP in alleviating inflammation and promoting tissue regeneration is achieved in approximately 1 month; therefore, all patients were scheduled to receive 4 treatment sessions administered at monthly intervals to achieve the best treatment outcome. Patients were allowed to stop the PRP treatment at any time-point If they felt satisfied with the results or if they were not willing to complete the full treatment course.

All patients were required to report a 3-day voiding diary and symptom inventory using the OSS, ICSI, and ICPI at each study visit. Data pertaining to FBC, daily urinary frequency, nocturia episodes, and VAS pain score were recorded at each study visit (baseline, 1, 2, 3 months, and 3 months after the 4th PRP treatment). Before each PRP injection and 3 months after the 4th PRP injection, patients were requested to report their current bladder condition. Uroflowmetry was performed and PVR assessed to measure the maximum flow rate (Qmax) and voided volume at baseline, at each post-treatment visit, and at 3 months after the 4th PRP injection.

The treatment outcome was assessed using the Global Response Assessment (GRA) at each time-point. The GRA is a seven-point symmetric scale that captures the patient’s general response to the treatment: markedly worse: − 3, moderately worse: − 2, slightly worse: − 1, no change: 0, slightly improved: + 1, moderately improved: + 2, and markedly improved: + 3^33^. The treatment result was considered to be excellent if patients reported an improvement in the GRA by ≥ 2 or if patients were free of bladder pain (VAS score = 0). Otherwise the treatment was considered as failure.

Urine samples were collected at baseline and at each study time-point for quantification of markers using a multiplexed immunoassay. A panel of 13 proteins including inflammatory markers, growth factors, cytokines/chemokines, and neurotrophic factors were chosen based on our previous proteomics study on the protein expression profiling in IC/BPS^24^. Markers of inflammation, angiogeneis, growth factors, and cytokines were measured using a multiplex, magnetic bead-based immunoassay (MILLIPLEX® MAP; EMD Millipore, Billerica, MA, USA) according to the manufacturer’s instructions. Data analysis was performed using the MILLIPLEX® Analyst v.5.1 software.

The measured urinary markers included VEGF, NGF, BNDF, TGF-β1, PDGF-AA, PDGF-AB, MMP-13, and MMP-1; the measured cytokines included IL-2, IL-6, IL-8, IL-1β, and TNF-α. Marker levels were measured at baseline and at the end-point at the fourth PRP injection (1 month after the 3rd PRP injection). The clinical parameters including pain VAS score, daily frequency, nocturia episodes, FBC, and GRA were also recorded and collated with the changes in urinary markers.

Differences between the urine protein levels at baseline and those at the post-PRP treatment time-points were assessed using the Mann–Whitney U test for non-parametric data. Changes in these markers were also collated with the improvement in GRA, VAS score, daily frequency, OSS, and FBC. All data are expressed as mean ± standard deviation. All analyses were conducted using SPSS for Windows (version 12, SPSS, Chicago, IL). Two sided *p* values less than 0.05 were considered indicative of statistical significance.
